# Cancer Immunotherapy and Next-Generation Clinical Immune Assessment

**DOI:** 10.3389/fonc.2014.00265

**Published:** 2014-09-24

**Authors:** Chao Ma, Rong Fan

**Affiliations:** ^1^Division of Physics, Mathematics and Astronomy, California Institute of Technology, Pasadena, CA, USA; ^2^David Geffen School of Medicine, University of California Los Angeles, Los Angeles, CA, USA; ^3^Department of Biomedical Engineering, Yale University, New Haven, CT, USA; ^4^Yale Comprehensive Cancer Center, New Haven, CT, USA

**Keywords:** cancer immunotherapy, immune suppression, tumor immunity, immune assessment, single cell analysis, cytokine, functional heterogeneity, combinatory therapy, immune evasion, functional proteomics

The interplay between cancerous cells and immune cells has always been an intriguing topic in medicine and biology. Cancer cells emerge from self-cells through a series of genetic mutations. They often retain self-cells’ capacity in being exempt from immune surveillance. Therefore, bringing cancer cells back under the radar of immune system has long been considered as a necessary step toward complete tumor eradication and long-term antitumor protection. Based on this rationale, a series of immunotherapies were designed and many have shown promising results. Some have gone through multiple stages of clinical trials. As a result, a successful immunotherapy is an intricate clinical procedure that affects the function of a myriad of cells. Only comprehensive studies that profile multiple aspects (e.g., cellular abundance, phenotypes, and functions) over time at the finest details can effectively monitor the convoluted immune response induced by therapy. Many recent technical developments aim to provide a solution for comprehensive clinical immune assessment.

In this book, we compiled a series of high-quality papers that summarize recent developments of immune assessment tool and methodology, as well as new biological findings in tumor immunity and cancer immunotherapy.

The book starts with a number of reviews and research articles that form an update of cancer immunotherapy. Ma et al. (1) reviewed new technologies to assess functional proteomics of single immune cells, their applications in clinical cancer immunotherapy, as well as new big-data computational methods to interpret the massive readouts. Next, a review paper by Chen et al. (2) highlighted recent advances in microfluidics tools used for functional immunophenotyping and emphasized the potential of integrated microfluidics circuitry. Klinke (3) focused on the concept of combining next-generation genome sequencing and computational power to uncover mechanism underlying tumor immunity evolution. In their opinion papers, Kwak et al. (4) and Fan et al. (5) hypothesized the importance of protein secretion profile in developing definitive correlates for cancer and immune heterogeneity.

The book goes on to the discussion of biology behind cancer immunotherapy. Monjazeb et al. (6) explored the topic of tumor induced immune suppression and proposed combinatorial therapy to induce antigen non-specific immune response and overcome immune evasion. Najjar and Finke (7) reviewed the role of myeloid derived suppressor cells (MDSC) in tumor mediated immune evasion and updated the status of pre-clinical and clinical tumor therapies designed for MDSC inhibition. Kawakami et al. (8) suggested that using combinatory therapy that targets shared immunosuppressive signaling pathway inhibitors to treat cancer. Dobrzanski (9) summarized recently discovered functions of CD4 T cell and new T cell lineages relevant to tumor immunity and tumor progression. Finally, in a research article, Milano et al. (10) showed pre-clinical evidence of nanocurcumin in improving the efficacy of dendritic cell-based immunotherapy for esophageal adenocarcinoma.

The editors thank all authors for their contributions and appreciate the valuable discussions with our reviewers. We wish that this special issue would serve as a reference book to the field and will inspire more thoughts and discussions for future investigation.

## Conflict of Interest Statement

The authors declare that the research was conducted in the absence of any commercial or financial relationships that could be construed as a potential conflict of interest.
